# Dtar-Finder: program for drug target identification and characterization in bacteria

**DOI:** 10.6026/97320630015209

**Published:** 2019-03-15

**Authors:** Purushottam Prasad, Rati Sudha

**Affiliations:** 1 P.G. Department of Zoology, ANS College, Magadh University, Patna (Barh) 803213, India

**Keywords:** Drug target, Perl script, C.botulinum

## Abstract

The drug target identification is the primary step for drug discovery. Recent development of computational techniques and availability of sequencing data has provided numerous opportunities for target identification but very few of them are fully automated. Here, we have developed a Perl program named Dtar-Finder for drug target identification and its characterization. Dtar-Finder predicts the drug targets which are essential to pathogen and non homologous to human, essential human anti-targets and gut microflora. This program is divided in 6 modules where modules 1-4 extract drug targets while module 5-6 predicts druggability and broad spectrum ability of identified candidates. The performance of this program in terms of sensitivity and specificity is calculated where specificity score was better compare to sensitivity score. Further, we have tested our script on C. botulinum (3572 proteins) and 35 potential drug targets have been identified. Out of which 16 broad spectrums candidates were predicted whereas 8 candidates are found to be druggable whiles remaining are considered to be 'novel'. These drug targets were cross-validated through literature showing 77.14% accuracy. Thus, the idea behind this work was to develop a fast, robust and generic program capable of finding drug targets in bacteria, which has been fulfilled satisfactorily.

## Background

Drug discovery is a vast process, which includes various stages and trials; no wonder even after the investment of huge amount of money and time pharmaceutical companies takes decades to launch a new product to the market [1]. Among all these stages a very primary stage is the drug target identification process under which we screen possible drug targets within a pathogen without harming the host; therefore for this a clear understanding of host-pathogen interactions is required. Human genome sequencing as well as sequencing of many pathogens in the recent years has produced a huge amount of data, which are quite useful for comparing both human and pathogenic at genomic level, and with the modern computational techniques this comparison can be done in no time [2, 3]. Thus filtering out huge number of protein has become much easier and convenient leaving very less number of proteins for wet lab cross validation saving both time and money.

There are many existing computational techniques for drug target identification known but only some are fully automated. T-iDT is one such potential drug target identification tool validated in Mycobacterium tuberculosis [4], which extracts drug targets, which are essential and non, homologous to human by comparing data with the Database of Essential Genes (DEG) [5] and human protein database. But it is stand-alone software and will not show updates of DEG database, which can only be done manually. Here, we have designed a Perl program named Dtar-Finder and fully automated the subtractive genomic approach for drug target identification in bacteria [6, 7, 8]. Dtar-Finder performs the following functions: 1) Identification of drug targets in bacteria 2) Characterization of the identified targets based on its druggability and broad-spectrum ability. This program is scripted under 6 modules where module 1- 4 identifies probable drug target candidates and module 5-6 characterizes the identified targets. The objective of this program is to not only to screen non-homologous human proteins (eliminates the chances of cross-reactivity of drug with similar human protein) and essential protein (required for bacterial survival) [9] but also filter out proteins homologous to anti-targets (human essential proteins) [10] and human gut microbiota (resides in gastrointestinal tract of healthy human) [11]. Special feature added in Dtar-Finder also characterized the identified targets on the basis of its druggability (homology search against Drugbank database) and broad-spectrum ability (homology search against pathogen database). Further, we have tested the performance of this userfriendly robust program.

## Methodology

### Perl code scripting and designing:

**Figure 1:**
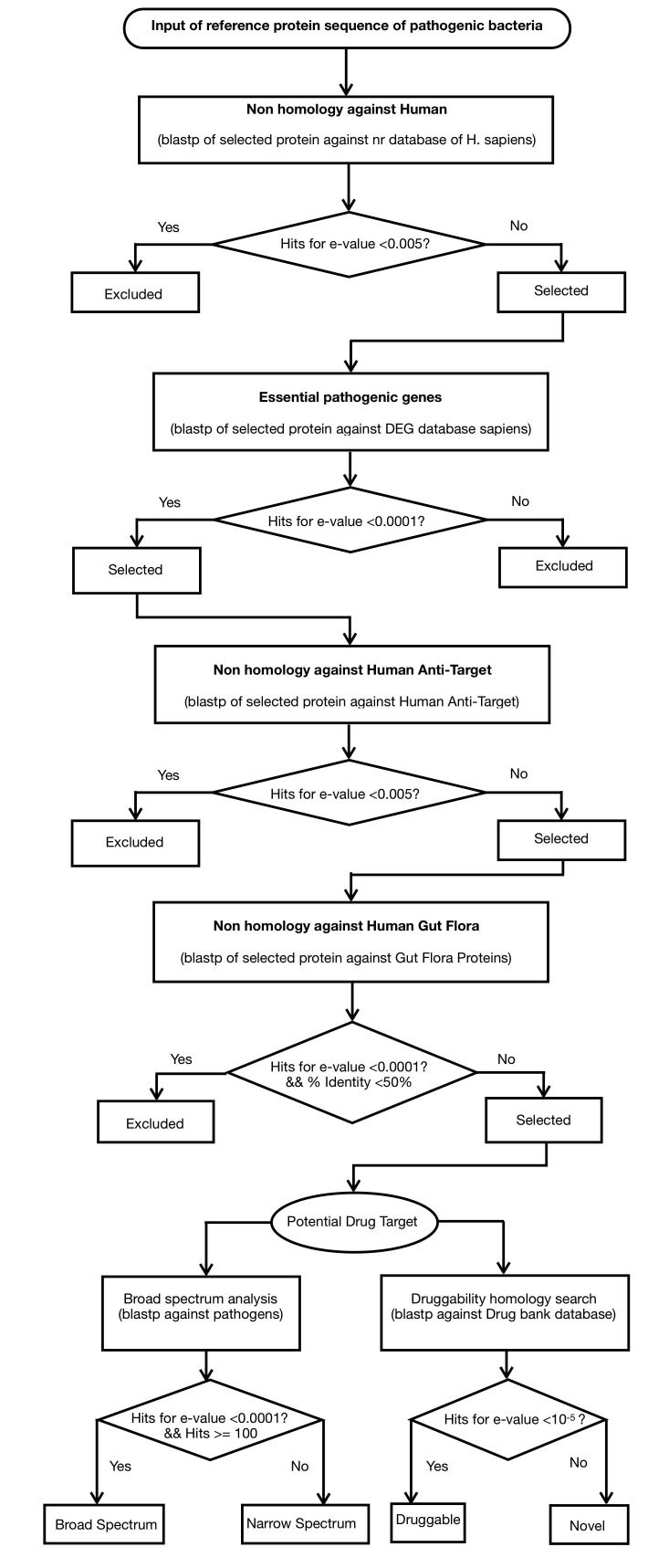
Flowchart representing the architecture of Dtar-Finder program for the identification of potential targets, druggability and broadspectrum ability in bacteria using subtractive genomics approach.

Dtar-Finder program (Dtar-Finder.pl) is available at https://gist.github.com/rati/ along with its instruction manual at https://gist.github.com/rati/ for the users. The overall workflow of Dtar-Finder is presented by Figure 1. This program is scripted in Perl language and runs under UNIX environment. As this code automatically runs Blastp program under set criteria of E-value and percentage identity therefore, NCBI-BLAST-2.2.25+ package was installed earlier into the system [12]. We have utilized several UNIX Shell commands required for trimming different files under this code. The protein files required were provided in fasta file format where each sequences starts with ">" (greater than symbol) with sequence id and description and entire protein sequences are kept in next single line which were manually achieved. Six databases has been downloaded and provided as reference files to the Blastp program and these are as follows: 1) non-redundant database of H. sapiens 2) essential genes from Database of essential genes 10.0 [5], 3) anti-targets (essential proteins) of a host (n=210), 4) human gut microbiota (n=66) [10], 5) targets from DrugBank 3.0 [13], 6) pathogen proteins (n=223) [10]. Dtar-Finder provides user-friendly environment where 1) User can customize BLAST criteria such as E-value and percentage identity etc 2) User can download and provide the program an updated version of database. The performance of the program was tested on 100 protein dataset (known drug target: non-drug target (50:50 ratio)) collected from literature and sensitivity (true positive rate) and specificity (true negative rate) of the output is calculated. Further, we have also predicted the potential drug targets and its characterization on 3572 reference protein sequences of C. botulinum (strain Hall/ ATCC 3502/ NCTC 13319/ Type A) [14].

Sensitivity (SN) = TP/(TP+FN)

Specificity (SP) = TN/(FP+TN)

 Where, TP= True positive; TN= True negative; FN= False negative and FP= False positive

## Output:

Dtar-Finder produces 3 result files (1) drug_targets.fasta - It carries potential drug targets ID and their corresponding sequence in the fasta format (2) drugability_result.fasta - It carries homologous drug bank targets (3) broad_spectrum_result - It carries the number of homologous pathogens against each target. The calculated sensitivity and specificity for the 100 protein dataset validates the performance of the program tabulated in Table 1 which clearly shows that the specificity score is having better results compared to sensitivity. Also, for 3572 proteins of C. botulinum, 35 potential drug targets have been predicted, out of which 16 drug targets were broad spectrums candidates and 8 drug targets were found to be druggable while remaining were seemed to be 'novel' targets (Table 2). These results were further cross-validated through literature and 27 drug targets with similar functions (functionality of 3 hypothetical proteins were predicted using INTERPROSCAN) [15] were found to be acting as a drug target in other bacteria whereas 8 targets did not show any result. That means the cross validation produced results with 77.14% of accuracy.

## Caveats:

Dtar-Finder provides results, which are fully computational, based and uses BLAST program for sequence similarity search hence; all the limitations related to this in-silico techniques are equally applicable to this script output. Here, we have collected reference files from different databases therefore all the limitations related with these databases are again applicable to our script result. Although the sensitivity and specificity scores are better but still needs further improvement. Also a detail investigation and wet lab validation of the result is required.

## Future development:

In future we can further include other qualitative characterization features such as cellular localization, functionality analysis of hypothetical proteins, etc in the later version of Dtar-Finder.

## Conflict of Interest

Authors declare no conflict of interest

## Figures and Tables

**Table 1:** Performance of Dtar-Finder against 100 protein dataset

Category	Sensitivity	Specificity
Drug targets	0.76	0.94
Druggability	0.92	1
Broad spectrum ability	0.86	0.9

**Table 2:** List of potential drug targets and qualitative characterization in C. botulinum using Dtar-Finder.

Target No.	NCBI ID	Gene Product Definition	Broad spectrum ability	Druggablility	Drugbank target
1	148378250	sensor histidine kinase	109(Yes)	Druggable	P52687, Q9X180
2	148378251	response regulator	120(Yes)	Druggable	P41789, P13632
3	148378307	AraC family transcriptional regulator	91(No)	Novel	-
4	148378313	iron compound ABC transporter permease	89(No)	Novel	-
5	148378314	iron compound ABC transporter permease	92(No)	Novel	-
6	148378317	iron dependent repressor	79(No)	Novel	-
7	148378526	Amino acid/polyamine transporter I	84(No)	Novel	-
8	148378616	rod shape-determining protein RodA	151(Yes)	Novel	-
9	148378703	AraC family transcriptional regulator	93(No)	Novel	-
10	148378747	LysM domain-containing protein	11(No)	Novel	-
11	148378755	TetR family transcriptional regulator	34(No)	Novel	-
12	148378828	RNA polymerase sigma-70 factor, ECF family	113(Yes)	Novel	-
13	148378842	GNAT family acetyltransferase	132(Yes)	Novel	-
14	148378872	transcription antiterminator	58(No)	Druggable	P39805
15	148378881	spore coat protein	64(no)	Novel	-
16	148378955	molybdenum ABC transporter permease	135(Yes)	Novel	-
17	148379137	ABC transporter permease	126(Yes)	Novel	-
18	148379139	Peptidase	100(Yes)	Novel	-
19	148379181	AraC family transcriptional regulator	105(Yes)	Druggable	P0A9E0
20	148379182	Major facilitator superfamily domain	44(No)	Novel	-
21	148379348	FUR family transcriptional regulator	147(Yes)	Novel	-
22	148379637	phage regulatory protein	1(No)	Novel	-
23	148380009	transferase, hexapeptide repeat family	152(Yes)	Druggable	P43886, Q0WKM4
24	148380045	DNA helicase	187(Yes)	Novel	-
25	148380184	oligopeptide transporter OPT family	43(No)	Novel	-
26	148380317	phage integrase	165(Yes)	Novel	-
27	148380349	Resolvase	96(No)	Novel	-
28	148380661	glycosyl transferase family protein	36(No)	Novel	-
29	148380668	glycosyl transferase family protein	77(No)	Novel	-
30	148380673	dTDP-4-dehydrorhamnose 3,5-epimerase	120(Yes)	Druggable	P26394, Q9HU21, O06330, O52806
31	148380679	3-deoxy-manno-octulosonate cytidylyltransferase	102(Yes)	Druggable	P44490, P42216
32	148380686	6-hydroxymethylpterin diphosphokinase MptE-like	19(No)	Novel	-
33	148380687	Glycosyltransferase	135(Yes)	Novel	-
34	148380997	alginate O-acetyltransferase AlgI	84(No)	Novel	-
35	148381302	diguanylate cyclase	83(No)	Druggable	Q9A5I5

## References

[1] Chan JN (2010). Trends Pharmacol Sci.

[2] Dutta A (2006). In Silico Biol.

[3] Sarangi AN (2009). J Comput Sci Syst Biol.

[4]  Singh NK (2006). In silico biol.

[5] Luo H (2014). Nucleic Acids Res.

[6] Reddy EH, Satpathy GR (2009). Online Journal of Bioinformatics.

[7] Katiyar A (2018). J Integr Bioinform.

[8] Sudha R (2019). Bioinformation.

[9] Sarkar M (2012). JMol Model.

[1] Raman K (2008). BMC Syst Biol.

[11] Neish AS, (2009). Gastroenterology.

[12] Altschul SF (1990). J Mol Biol.

[13] Knox C (2011). Nucleic Acids Res.

[14] Pruitt KD (2007). Nucleic Acids Res.

[15] https://www.ebi.ac.uk/interpro/.

